# Supplementary material for: The Potential of the Synthetic Strigolactone Analogue GR24 for the Maintenance of Photosynthesis and Yield in Winter Wheat under Drought: Investigations on the Mechanisms of Action and Delivery Modes
Source: Plants (Basel). 2021 Jun 16;10(6):1223. doi: 10.3390/plants10061223 (PMC8233996; doi:10.3390/plants10061223)
Supplement: Supplementary file 1 [file plants-10-01223-s001.zip › plants-1257684-supplementary.pdf]

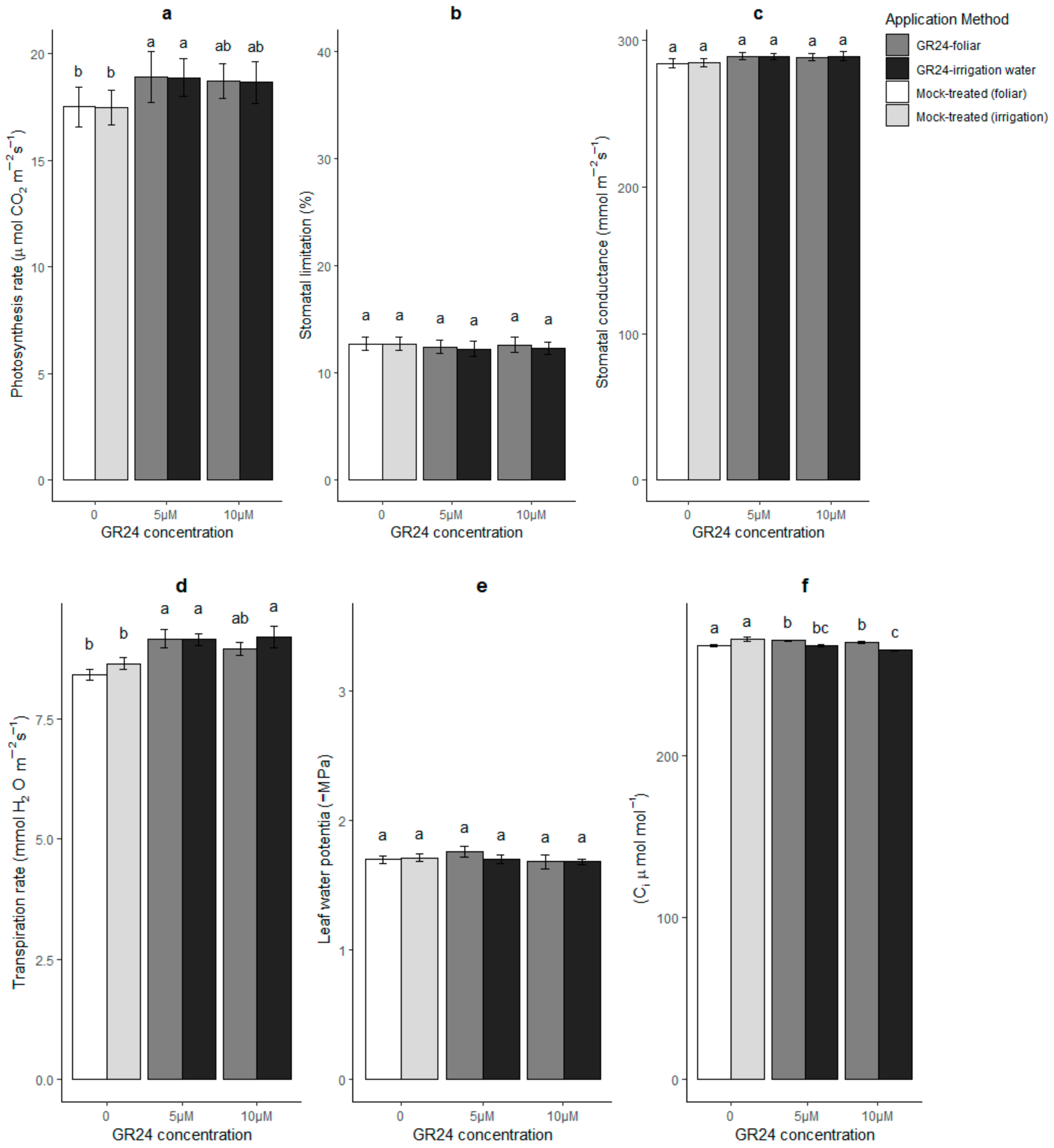

Figure S1. Photosynthesis rate (a), stomatal limitation (b), stomatal conductance (c), transpiration rate (d), leaf water potential (e) and substomatal CO2 levels (Ci) (f) in wheat plants in response to two methods of GR24 application under irrigated conditions. Mock-treated plants received a water and acetone solution. Each value represents the mean ± SE (n = 4, each replicate the pool of four plants). Different letters on top of bars indicate significantly different means for p ≤ 0.05 (LSD test).

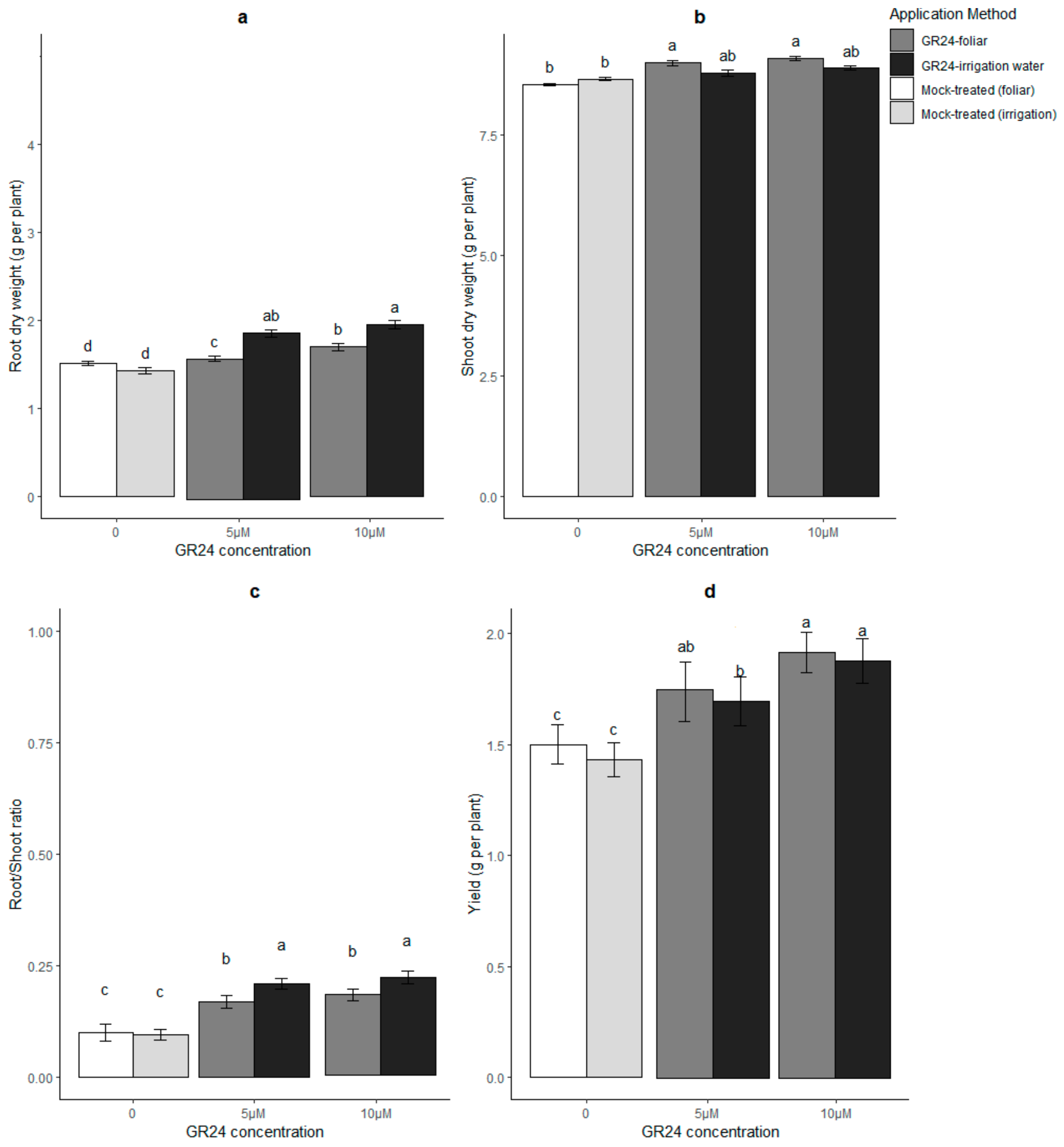

Figure S2. Root dry weight (a), shoot dry weight (b), root/shoot ratio (c), and yield (d) in wheat leaves in full irrigation condition, in response to two application methods for GR24. Mock-treated plants received a water and acetone solution. Each value represents the mean ± SE (n = 4, each replicate the pool of four plants). Different letters on top of bars indicate significantly different means for $p \leq 0.05$ (LSD test).

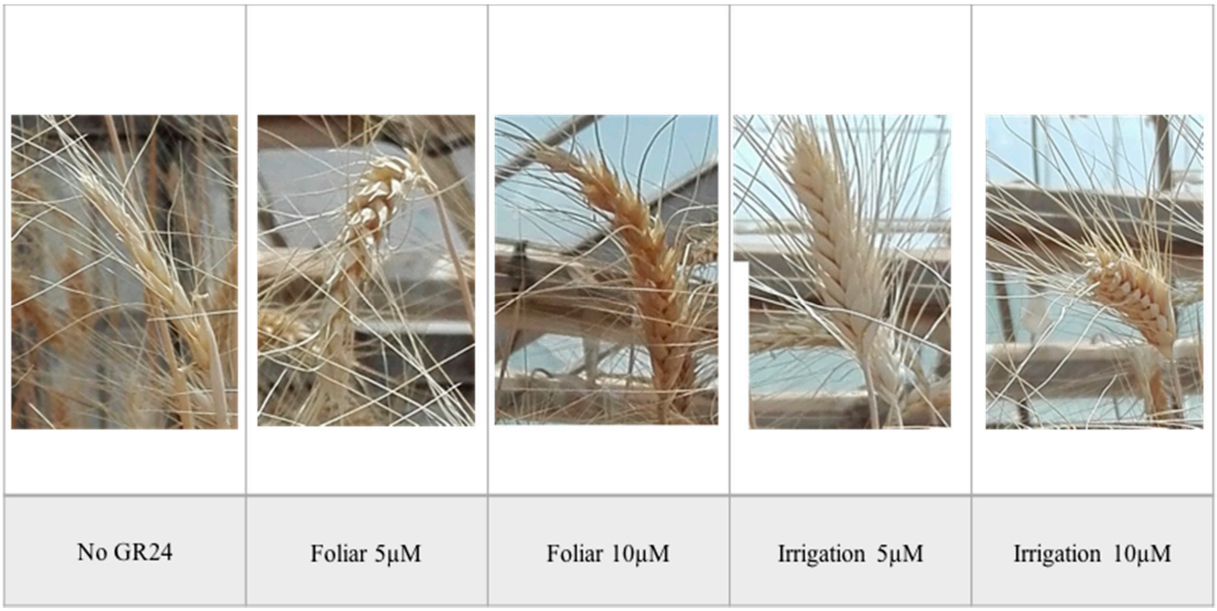

Figure S3. Spike appearance at maturity.

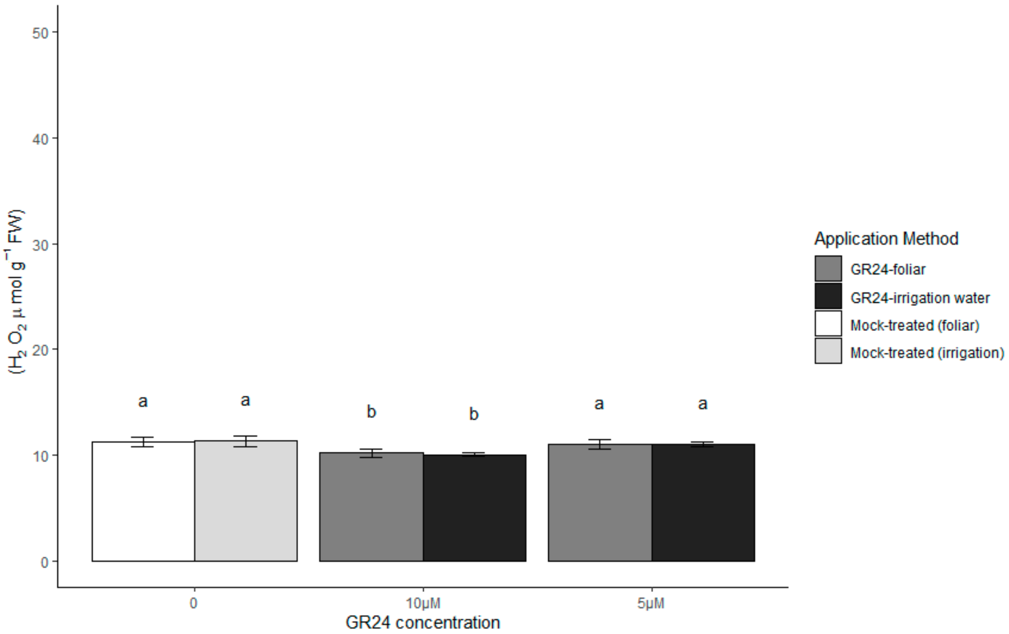

Figure S4. H_2O_2 in wheat plants in response to two methods of GR24 application in full irrigation conditions. Mock-treated plants received a water and acetone solution. Each value represents the mean ± SE (n = 4, each replicate the pool of four plants). Different letters on top of bars indicate significantly different means for $p \leq 0.05$ (LSD test).

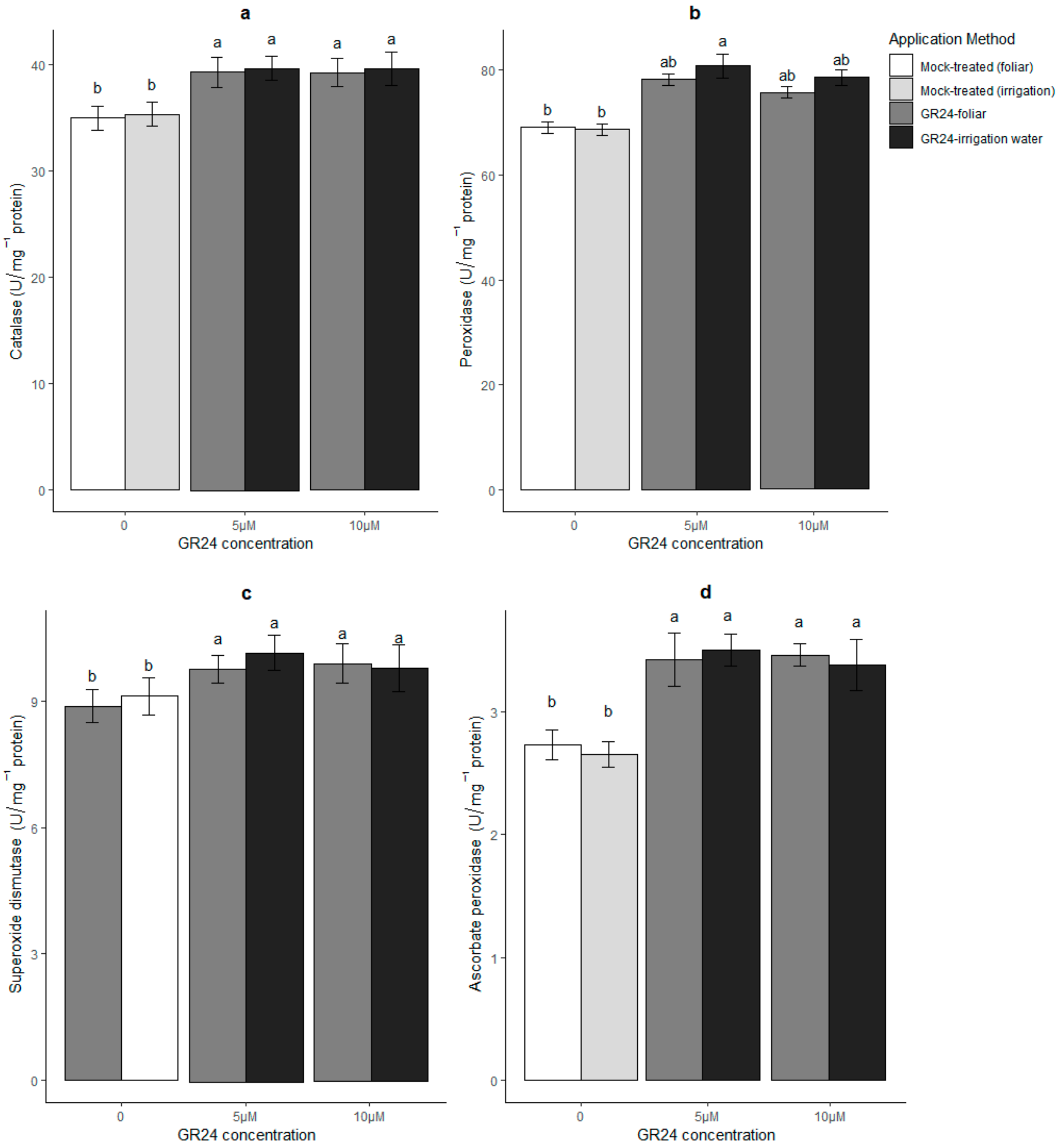

Figure S5. Catalase (a), peroxidase (b), superoxide dismutase (c) and ascorbate peroxidase (d) enzyme activities in wheat leaves in full irrigation conditions, in response to two application methods for GR24. Mock-treated plants received a water and acetone solution. Each value represents the mean ± SE (n = 4, each replicate the pool of four plants). Different letters on top of bars indicate significantly different means for $p \leq 0.05$ (LSD test).
